# Extended performance analysis of deep-learning algorithms for mice vocalization segmentation

**DOI:** 10.1038/s41598-023-38186-7

**Published:** 2023-07-11

**Authors:** Daniele Baggi, Marika Premoli, Alessandro Gnutti, Sara Anna Bonini, Riccardo Leonardi, Maurizio Memo, Pierangelo Migliorati

**Affiliations:** 1 Department of Information Engineering, University of Brescia, Brescia, Italy; 2 Department of Molecular and Translational Medicine, University of Brescia, Brescia, Italy

**Keywords:** Neuroscience, Computer science

## Abstract

Ultrasonic vocalizations (USVs) analysis represents a fundamental tool to study animal communication. It can be used to perform a behavioral investigation of mice for ethological studies and in the field of neuroscience and neuropharmacology. The USVs are usually recorded with a microphone sensitive to ultrasound frequencies and then processed by specific software, which help the operator to identify and characterize different families of calls. Recently, many automated systems have been proposed for automatically performing both the detection and the classification of the USVs. Of course, the USV segmentation represents the crucial step for the general framework, since the quality of the call processing strictly depends on how accurately the call itself has been previously detected. In this paper, we investigate the performance of three supervised deep learning methods for automated USV segmentation: an Auto-Encoder Neural Network (AE), a U-NET Neural Network (UNET) and a Recurrent Neural Network (RNN). The proposed models receive as input the spectrogram associated with the recorded audio track and return as output the regions in which the USV calls have been detected. To evaluate the performance of the models, we have built a dataset by recording several audio tracks and manually segmenting the corresponding USV spectrograms generated with the Avisoft software, producing in this way the ground-truth (GT) used for training. All three proposed architectures demonstrated precision and recall scores exceeding $90\%$, with UNET and AE achieving values above $95\%$, surpassing other state-of-the-art methods that were considered for comparison in this study. Additionally, the evaluation was extended to an external dataset, where UNET once again exhibited the highest performance. We suggest that our experimental results may represent a valuable benchmark for future works.

## Introduction

Mice produce ultrasonic vocalizations (USVs) with a frequency from 30 kHz to 110 kHz to communicate one another during their lifespan^1^. In particular, pups emit USVs during separation from the mother and the littermates to induce maternal caregiving behavior^2,3^. Also juvenile mice vocalize, especially during play and social interaction^4,5^. In addition, adult mice emit USVs in social context such as courtship, mating and social investigation. The better described adult USVs are those produced during male-female interactions with an important role in social/sexual behaviors^6^. Also female mice emit USVs when they interact with other females, usually associated with a high level of social investigation of the female stimulus to establish affiliative relationships^7,8^.

Recently, the relevance of USVs has been consolidated as a valid tool for behavioral analysis of mice in both the context of ethological studies and in the field of brain pathologies, especially those characterized by deficit in communication such as neurodevelopmental disorders (NDDs) and in particular autism spectrum disorders (ASD)^9–11^. Altered calling pattern are found in several mouse models of NDDs and ASD^12,13^. So, USVs study can be used as a useful tool to analyze the mechanisms at the basis of these neuropsychiatric disorders and to test the effects of pharmacological treatments for these pathologies^14^.

USVs can be acquired with an ultrasound sensitive microphone and later analyzed on the basis of quantitative parameters using specific softwares (e.g., SASLab by Avisoft Bioacoustics, Metris Sonotrack and Noldus UltraVox XT). Unlike the quantitative analysis of the USVs, which is carried out automatically by the software, the qualitative analysis is more complex and often still requires the intervention of the operator (manual analysis). Ultrasonic calls can be classified based on different criteria. For examples, Scattoni’s classification, that is one of the most used, is based on internal sound frequency changes, duration and shape in time-frequency domain, and it is composed of 10 categories of calls such as: complex, harmonics, two-syllables, upward, downward, flat, chevron, short, composite and frequency steps^9^. In the literature, other methods of qualitative classification have been proposed and each one uses different types of calls^15,16^. In all cases, these systems require a manual classification of calls that is highly time-consuming and is also operator-dependent (high variability).

Interestingly, in the last five years some automated classification systems have been developed. They can be distinguished between two groups based on the approach adopted for their implementation. Indeed, some schemes employ pure signal processing methods, which rely explicitly on the acoustic parameters of the recordings^17–20^. Other systems, instead, exploit machine learning algorithms and neural networks for a faster and objective identification of spectrogram features and also a standardization of USVs analysis in comparison of the manual classification of calls^21–25^.

Independently of the approach, the classification performance of an automated classification system is strongly affected by its ability of initially segmenting with high precision all the USVs present in the recording, namely, detecting the time frames that enclose the start and the end of a vocalization. Thus, the USV segmentation represents a crucial step for the general framework, and the more it is accurate, the more the following processing steps are reliable. Even if the aforementioned systems have achieved remarkable results, no deep research investigation involving the newest deep learning methods has been carried out for this task.

In this paper, we investigate the performance of different supervised deep learning algorithms for automated USVs segmentation. In particular, we design and compare the performance of 3 distinct architectures, which represent three popular methods for segmentation task: an Auto-Encoder Neural Network^26^ (AE), a U-NET Neural Network^27^ (UNET) and a Recurrent Neural Network^28^ (RNN). By comparing these three methods, we can gain a direct insight into the achievable performance in the field of USV segmentation. They generally allow to exploit both temporal correlation of audio signals (in particular, the RNN) and spatial correlation of images (in particular, the AE and the UNET). This fact leads them to be particularly suitable for working on 2-D spectrograms, as in this work. The final configuration of the proposed architectures has been chosen with the objective of reaching significant results and, at the same time, guaranteeing a particularly low computational complexity.

We carry out extensive experiments to accurately evaluate the performance of the proposed systems. For the assessment, we adopt the Intersection over Union (IoU) metric, which calculates the overlap between the predicted USV and the ground truth USV, normalized by the total area covered by the two segmentations. Since our goal is to detect objects - specifically, USV calls - such metric is the most appropriate for evaluating the segmentation effectiveness of a method.

Furthermore, our analysis includes a comparison with various software tools used for USV segmentation in mice. These include DeepSqueak^22^ and HybridMouse^25^, which employ deep learning methods, as well as A-MUD^18^ and USVSEG^19^, which rely on traditional signal processing techniques. We will show that our proposed methods offer superior segmentation performance, even though they require less complexity and do not necessitate any parameter settings. The comparison is performed on both our dataset, which has been constructed by capturing multiple audio tracks and manually segmenting the corresponding USV spectrograms generated using Avisoft software, and an external dataset. Our results represent a valuable benchmark for future works and the analysis model is freely available to all researchers working in the field of mice communication analysis. We have also publicly shared our dataset, which includes both our recorded audio tracks and experts’ annotations.

## Materials

### Animals

40 control wild-type (WT) pups (B6;129PF2) and 39 NF-$\kappa$B p50 knock-out (KO) pups (B6;129P2-Nf$\kappa$b 1tm 1 Bal/J) have been used for our experiment. These animals have been obtained by mating of WT and KO mice breeders purchased from The Jackson laboratories (Bar Harbor, ME, USA) and housed in standard cages in our animal facility kept at $23\pm 2 ^\circ$C of temperature and $55\pm 15\%$ of humidity in a 12 hours light/dark cycle with food and water ad libitum. Behavioral procedures have been performed according to the ARRIVE guidelines and European Communities Council Directive guidelines (CEE $\hbox {N}^\circ$ 86/609), and approved by the Animal Welfare Body (OPBA) of the University of Brescia and by the Italian Ministry of Health.

### Maternal separation-induced USVs recording

Each pup has been separated from the mother and the litter and then introduced into an empty glass container for 3 minutes. An ultrasound sensitive microphone (UltraSoundGate Condenser Microphone CM 16, Avisoft Bioacoustics, Berlin, Germany) has been placed 20 cm above the container to record USVs emitted by pup at postnatal day (PND) 6, 8 and 12 as previously described^13,24,29^. Briefly, the recordings have been performed 3 minutes along by the Avisoft software (Avisoft Bioacoustics, Berlin, Germany), which collects the data streams from the microphone (sampling frequency equal to 250 kHz, 16 bit per sample), it saves them into an audio file and returns a real-time spectrogram in order to allow the operator to monitor the presence of calls during the recording. To generate the spectrogram of the segments, a step size of 8 samples and 512 Fourier coefficients have been used. A kaiser window has been applied, with a length matching the number of Fourier coefficients and a beta value of 5.

### Dataset creation

At the end of the recording stage, 198 pup mice three minutes long audio tracks have been collected. The associated spectrograms $S_i$ compose the dataset, with $i=1,\ldots ,198$. Our experts have then manually segmented the USVs, by identifying 49, 750 USVs present in all the spectrograms. Thus, a label vector $y_i$ is associated with the *i*-th spectrogram, the elements of which indicate the presence or the absence of a USV for each time frame of the spectrogram. Each label can assume either the value 1 when a USV corresponding to that time instant has been manually detected by the operator, or the value 0 when no USV has been identified. In total, there are 911, 120, 892 samples corresponding to the USV class and 1, 471, 342, 084 samples related to the noise class. The entire dataset has been finally randomly split into a training set and a test set in a $4\!:\!1$ proportion, so 158 and 40 spectrograms with related label vectors compose the training and test sets, respectively.

**Figure 1:**
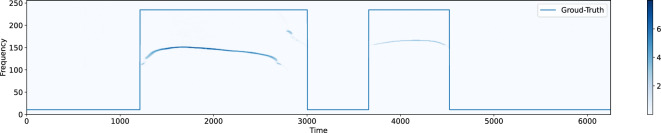
An example of USV segmentation.

## Proposed methods

The aim of this work is to automatically segment USVs (see Fig. 1 as an example). We approach this task by proposing and comparing 3 distinct deep-learning architectures: an Auto-Encoder Neural Network (AE), a U-NET Neural Network (UNET) and a Recurrent Neural Network (RNN). Ideally, each model would take as input a spectrogram $S_i$ with size $(M\!\times \!N)$ and would return a $(1\!\times \!N)$ binary vector $y^{(p)}_i$, that would indicate whether a USV is present for each time frame. However, the training process is too expensive from a computational point of view if the entire spectrogram is used as input. Thus, each $S_i$ is subdivided into shorter temporal length $(M \times n)$-spectrograms $s_{ij}$ with an overlap equal to the $50\%$ of the segment length, and $n=2^{11}$, namely, 65.536 ms.

Thus, each model takes an $(M \times n)$-spectrogram $s_{ij}$ as input and returns an *n*-length binary vector $y_{ij}^{(p)}$ as output, where the label $y_{ij}^{(p)}[{\bar{k}}]$ indicates whether a USV occurs at the corresponding time frame $s_{ij}[{\bar{k}},f]$, independently of the frequency *f*. Then, all the binary vectors $y^{(p)}_{ij}$ associated with the spectrograms $s_{ij}$ are processed for generating the final label vector $y^{(p)}_i$. Since $S_i$ is divided into $50\%$ overlapping $s_{ij}$, the second half of $y^{(p)}_{ij}$ matches with the time frames of the first half of $y^{(p)}_{i(j+1)}$. In order to determine the final labels of those time frames, we perform the element-wise OR logical operation between the corresponding positions. The resulting labels are thus concatenated for obtaining $y^{(p)}_i$. The final segmentation of a USV call is identified with its starting and end point. The starting point is determined by a transition from 0 to 1 in $y^{(p)}_i$, while the consecutive transition from 1 to 0 represents the end of that vocalization.

**Figure 2:**
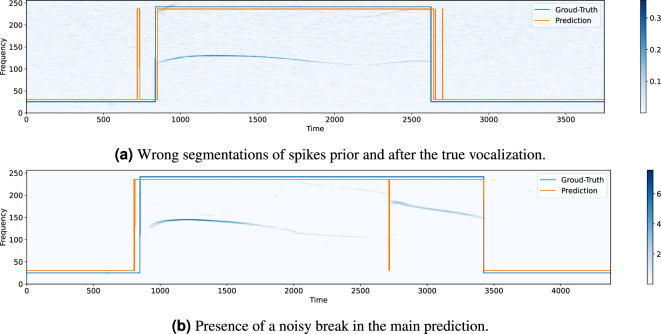
Two examples of inconvenient situations emerging from a not post-processed segmentation. (**a**) some spikes are incorrectly detected prior and after the USV, while in (**b**) the main prediction is split into two regions because of an extremely short break inside. Both cases can be properly fixed by applying a post-processing algorithm based on mathematical morphology on the output of the models.

However, this association may be too rigid, since the models return as output the classification of each single time frame. Thus, the wrong detection of isolated positives may lead to identify extremely short USVs which, however, cannot clearly represent a true detection. For example, in Fig. 2a, it is shown the output of a model (orange signal) for a given spectrogram, which is compared with the true USV call (blue signal). It can be noted that there are some spikes outside the main prediction, which are seemingly not significant and that negatively affect the segmentation output. Another inconvenient situation is depicted in Fig. 2b, where a short interruption creates two distinct USVs. Also in this case, it would be appropriate consider that there exists just one single prediction, which would be obtained by connecting the two predictions.

**Figure 3:**
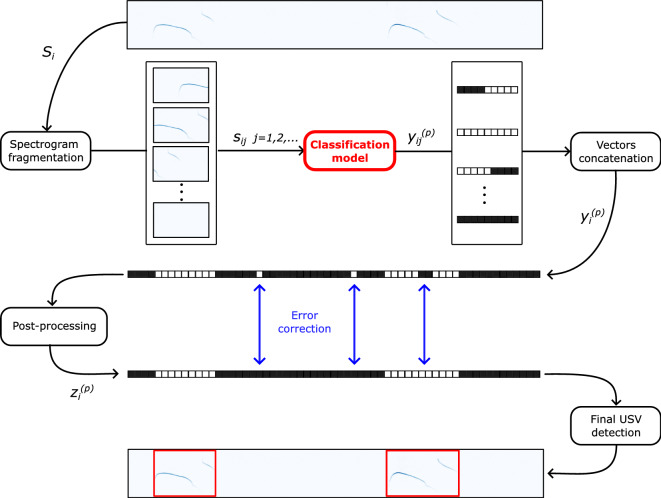
General framework for the USV segmentation. The spectrogram $S_i$ associated with an entire audio track is divided into shorter fragments $s_{ij}$. A classification model takes the fragments separately as input and returns for each of them a binary vector $y_{ij}^{(p)}$, the elements of which indicate if a USV is present at the corresponding time frames (white square) or not (black square). The vectors are then concatenated, generating the vector $y^{(p)}_i$ associated with $S_i$. Finally, a post-processing algorithm allows to remove potential isolated spikes or fill undesired small interruptions, generating this way $z^{(p)}_i$. For the sake of visibility, the squares associated with the single time predictions are drawn larger with respect to the time resolution of the depicted spectrogram.

Of course, the presence of noisy spikes or inconvenient breaks strongly affects the segmentation performance of the models because of the increase of false positives and, potentially, false negatives. For this reason, we propose to apply a post-processing algorithm based on mathematical morphology on the output of the models, in order to correct such issues. The algorithm consists in implementing the *closing* and the *opening* morphological operators on the label vector $y^{(p)}_i$ associated with the entire spectrogram $S_i$. We recall that the *closing* operation is defined as the sequence of the *dilation* and *erosion* basic morphological operators, while the *opening* operation is the dual counterpart, and it is defined as the sequence of the *erosion* and *dilation* operations. In this way, we are able to fill undesired adjacent regions of zeros potentially present in our binary prediction and, at the same time, remove undesired adjacent regions of ones. We name $z^{(p)}_{i}$ the resulting post-processed label vector. Summarizing, we depict the overall processing framework in Fig. 3.

**Figure 4:**
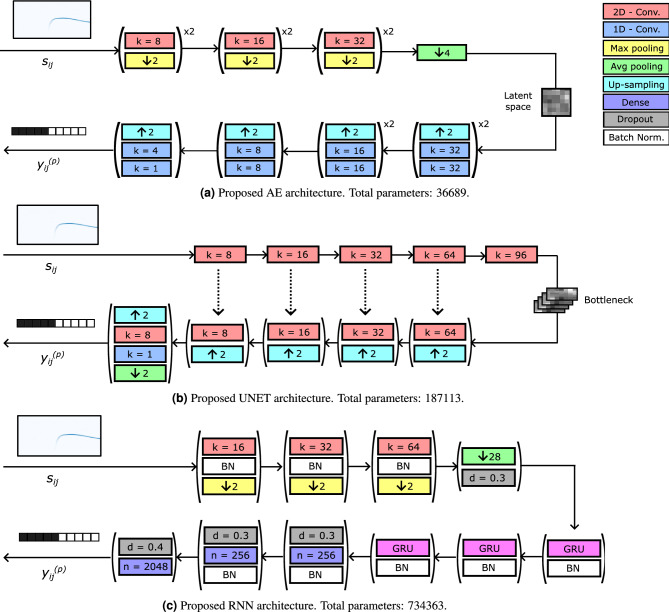
Flowchart of the three proposed models. The meaning of the blocks for all the 3 architectures is reported in the legend in (**a**). *k*, *n* and *d* specify the number of filters, the number of neurons and the dropout rate for the convolutional, dense and dropout layers, respectively.

Figure 4 reports the proposed architectures corresponding to the 3 classification models investigated in this work. The final configuration of those architectures has been achieved by performing several trials and tests over the training set. In particular, we have evaluated the performance at varying of the complexity of the models, by modifying the number of layers and the number of filters, neurons and time units in the convolutional, dense and recurrent layers, respectively. Remarkably, the final architectures, which are presented in detail in the following, allow to reach high performance and, at the same time, they are characterized by a relatively small number of parameters, thus requiring a particularly low computational complexity.

### First model–auto-encoder neural network

An Auto-Encoder Neural Network is a particular artificial neural network architecture that is largely used for segmentation of 2-D data, as, for example, images. The architecture is composed of two main parts: in the first, an encoder squeezes the information stored in the input data through some filters (convolutional layers) in a third dimension, while reducing the first and second dimensions. In the second stage, a decoder uses the information extracted by the encoder and stored in the third dimension to reconstruct the first and second dimensions, in accordance to the expected output. Also the decoder is typically composed of convolutional layers, interleaved by up-sampling layers. Based on the task, the output can be either a reconstructed version of the original image or provide for a classification map (e.g., a binary mask as in this case).

The proposed AE architecture is illustrated in Fig. 4a. In the current implementation, the encoder is composed of 6 convolutional layers with 8, 8, 16, 16, 32 and 32 filters, respectively, each of them adopting a Rectified Linear Unit (ReLU) activation function and initialized with a He normal kernel. Each convolutional layer is followed by a maximum pooling layer which reduces both dimensions by a factor of 2. Then, an average pooling layer removes one dimension from the feature space by computing the mean over the frequency axis, finally generating the latent space. Next, the decoder is composed of 6 1-D up sampling layers with sampling factor equal to 2. These layers compensate the max-pooling factor applied at the encoder and allow to increase the size of the time dimension to reach the original temporal resolution of the spectrogram. Each of them is followed by two 1-D convolutional layers with (32, 32), (32, 32), (16, 16), (16, 16), (8, 8), (4, 1) filters, respectively. The last convolutional layer adopts a sigmoid activation function which returns a real value between 0 and 1 for each time frame of the spectrogram, that represents the prediction confidence and determines the final output of the model.

### Second model– U-NET neural network

As the Auto-Encoder, also the U-Net Neural Network is an artificial neural network architecture widely used for 2-D segmentation. While the structure of the two architectures is similar, the main difference lies to the fact that each component of the encoder exchanges information with the corresponding component of the decoder. In particular, the output of a given layer at the encoder is used as input by the corresponding level at the decoder, where it is concatenated with the output resulting from the previous layer at the decoder.

The proposed architecture in the present context is depicted in Fig. 4b. The encoder is composed of 5 convolutional layers with 8, 16, 32, 64, and 96 filters, respectively, each of them using a ReLU activation function and initialized with a He normal kernel. Each layer performs the convolution with a kernel stride equal to 2 in both directions, reducing in this way both dimensions through the levels. Instead, the decoder is composed of 4 sets of layers, each of them consisting of an up-sampling layer with factor 2 along both the two dimensions and a convolutional layer with 64, 32, 16, and 8 filters, respectively. The kernel size is fixed at 3*x*3. Each output from the convolutional layer is concatenated with the output of the corresponding convolutional layer at the encoder. Next, an additional up-sampling layer is implemented in order to reach the final temporal resolution equal to the one of the input spectrogram, followed by two more convolutional layers with 8 and 1 filters, respectively. The last one adopts a sigmoid activation function, which returns the output of the model associated with each time frame of the spectrogram. Note that in the AE architecture the dimensionality reduction is performed in the bottleneck between the encoder and the decoder. On the contrary, in the UNET the first dimension is eliminated just at the end of the network, through an average pooling applied on the first dimension. In this way, it is possible to perform a concatenation with coherent data between the encoder and the decoder.

### Third model–recurrent neural network

A Recurrent Neural Network (RNN) is an artificial neural network that is typically used with time series or sequence data. It works with a memory element that is able to store the state of a previous input and use it to produce the next output. The RNN can work in 4 principal mode: the one-to-one mode, in which a single output is produced for each input; the one-to-many mode that returns one output per each sequence element for each new input element (e.g., used in music generation); the many-to-many mode that assigns an output to each element of the sequence in input (e.g., used for translations); and the many-to-one mode that returns a single element for each sequence in input (e.g., used for next element prediction). The RNN layers mostly used in order to introduce the recurrence in the network are Long Short-Term Memory (LSTM) and Gated Recurrent Unit (GRU). The LSTM uses three gates called input, output, and forget gate. These gates determine which information needs to be retained for future predictions. Instead, the GRU uses a reset and update gate for selecting the important information.

The proposed architecture is shown in Fig. 4c. The first part allows to extract the features from the input data and it is composed of three subsets of layers. All the sets contain a convolutional layer with 16, 32 and 64 filters, respectively. The first adopts a $3 \times 3$ kernel, while the next ones a $5 \times 5$ kernel. The convolutional layers of all the sets are followed by a batch normalization layer, which acts as regularizer, a ReLU activation function and a maximum pooling layer that reduces the tensor size by a factor of 2 along both the dimensions. Next, a dimensionality reduction from 3 to 2 is performed by an average pooling layer, which computes the mean over the frequency axis and returns one vector corresponding to the time axis of the spectrogram. Such time sequence is then passed to a cascade of recurrent layers consisting of a GRU mechanism with 32, 16, and 1 time units. In these layers, the correlation of each time sample with the temporal neighbors is exploited for extracting the relevant information, which is stored in a new tensor dimension. The last recurrent layer has one time unit for finally reducing the new dimension to 1. Then, a dropout layer contributes to randomly discard some network parameters, in order to prevent overfitting.

The second part of the network is composed of 3 fully connected (dense) layers with 256, 256 and 2048 neurons, respectively. The dimension of the third dense layer corresponds to the number of time frames of the input data, in order to obtain the final classification for each time frame of the spectrogram. The first and the second dense layers are followed by a batch normalization layer for regularization purpose and a ReLu activation function. As for the AE and the UNET architectures, the last dense layer is followed by a sigmoid activation function. It has been found beneficial to include also three dropout layers with a drop factor equal to 0.3, 0.4 and 0.4, respectively, for reducing overfitting.

### ARRIVE guidelines

The present study is reported in accordance with ARRIVE guidelines.

## Experimental results

In this section, the performance of the proposed architectures are presented. A *k*-fold cross validation has been performed over the training set for determining the optimal hyperparameters for each model, with $k=5$. A binary cross-entropy has been adopted as loss function. The Adam optimizer with learning rate in the ranges from $5\cdot 10^{-5}$ to $10^{-7}$, from $10^{-4}$ to $10^{-6}$ and from $10^{-4}$ to $10^{-7}$ has reported the best performance for the RNN, AE and UNET, respectively, when considering a maximum number of epochs equal to 100 and setting an early stopping with patience equal to 10 epochs for all the architectures. According with the obtained hyperparameters, the models have been thus trained over the entire training set and evaluate over the independent test set.

**Figure 5:**
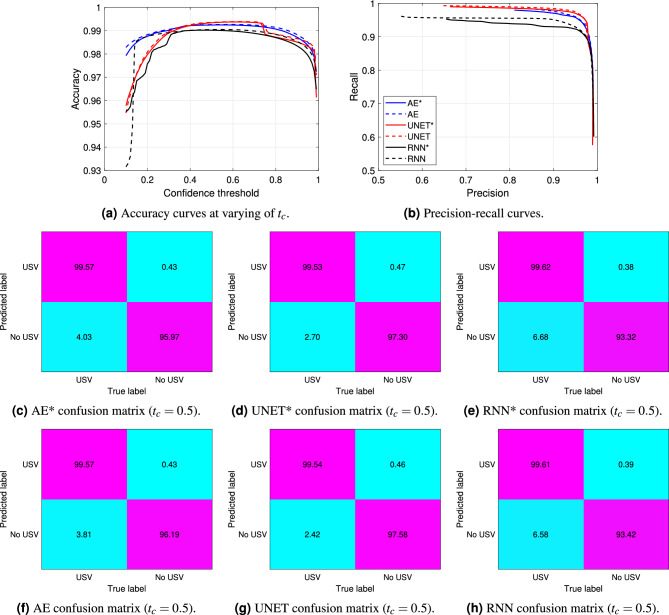
Performance comparison between the models in terms of time frame classification, with and without taking into account the post-processing algorithm. (**a**) and (**b**) show the accuracy and precision-recall curves, respectively. (**c**-**e**) depict the confusion matrices associated with the models without post-processing. (**f**–**h**) depict the confusion matrices associated with the models when implementing post-processing.

For reference, the mean and variance computation times to segment an entire spectrogram associated with a 3-minutes length audio track on a desktop computer (AMD Core Ryzen 9, 128GB RAM, graphic card NVIDIA GeForce RTX 3090 with 24 GB RAM) are $25.138 \pm 0.842$, $24.758 \pm 0.540$ and $49.231 \pm 0.922$ seconds for AE, UNET and RNN, respectively, using JupyterLab 3.3.2 with Python 3.8.10 and Tensorflow 2.8.0.

### Time frame classification

First, we evaluate the performance of the proposed models to correctly detect the presence of a USV for each time frame of a spectrogram, thus without considering the entire USV detection. Of course, even this initial assessment is performed on the test set. So, we compare the ground-truth (GT) label vectors $y_{ij}$ with the predicted label vectors $y^{(p)}_{ij}$ and $z^{(p)}_{ij}$. For the assessment, we calculate the precision, recall and accuracy metrics, which are defined as follows:

1 $$\begin{aligned} \text {precision} = \frac{\text {TP}}{\text {TP}+\text {FP}}; \quad \text {recall} = \frac{\text {TP}}{\text {TP}+\text {FN}}; \quad \text {accuracy} = \frac{\text {TP}+\text {TN}}{\text {TP}+\text {TN}+\text {FP}+\text {FN}}. \end{aligned}$$

We explicit that when $y_{ij}[{\bar{k}}]=1$ then $y^{(p)}_{ij}[{\bar{k}}]=1$ represents a True Positive (TP), while $y^{(p)}_{ij}[{\bar{k}}]=0$ is a False Negative (FN). Similarly, when $y_{ij}[{\bar{k}}]=0$ then $y^{(p)}_{ij}[{\bar{k}}]=1$ represents a False Positive (FP), while $y^{(p)}_{ij}[{\bar{k}}]=0$ is a True Negative (TN).

The results are illustrated in Fig. 5, in which the dashed and solid lines refer to the methods with and without the implementation of the post-processing algorithm, respectively. To ensure clarity, we will use the symbol * to refer to the model performance without post-processing from this point onward. In particular, Fig. 5a shows the comparison between the accuracy curves of the three examined models. The accuracy points are computed at varying of the confidence threshold $t_c$ reported in the *x*-axis, which is set in the last layer of the models for determining the predicted binary labels. The same $t_c$ values are used to compute the Precision-Recall points in Fig. 5b. Even if the solid curves associated with the results without post-processing are extremely high, the dashed lines still indicate a slight performance improvement. This remarks the benefit of implementing the morphological operators on the output of the models, which, however, will be more evident when analyzing the results for the USV segmentation. As a further comparison, Fig. 5c–h display the corresponding confusion matrices when $t_c$ is set to 0.5. These matrices highlight the balanced performance of the models in accurately classifying both classes.

### USV segmentation

To evaluate the segmentation quality of the models, we compute the Intersection over Union (IoU) measure associated with each USV detection. Such metric returns a value between 0 and 1 that represents the area of overlap between the predicted segmentation and the GT divided by the area of union between the predicted segmentation and the ground truth. Thus, a predicted segmentation is considered a TP when it intersects a GT such that the resulting IoU is greater than a given threshold $t_{\text {IoU}}$, otherwise it is identified as a FP. Also, we increase the FN when a true USV call does not intersect any predictions such that the corresponding IoU is greater than $t_{\text {IoU}}$.

**Figure 6:**
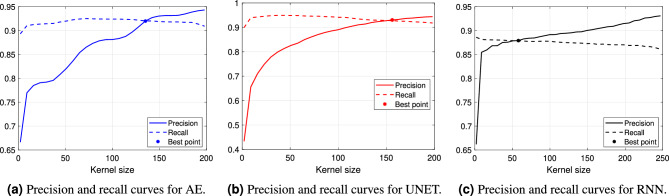
Training vocalization segmentation performance of the models at varying of the kernel size used in the post-processing.

First, in order to determine the optimal set-up of the post-processing algorithm, several 1-D kernel sizes have been tested for each model over the training set. Fig. 6 shows the performance at varying of the kernel length. The intersection point between the precision and the recall curves is selected as optimal value. Thus, kernels composed of 135, 150 and 65 samples are finally used for AE, UNET and RNN, respectively, on the test set.

**Figure 7:**
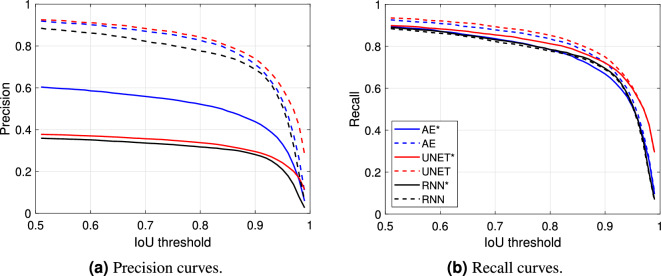
Precision and recall curves of the models at varying of the IoU threshold with and without post-processing.

**Figure 8:**
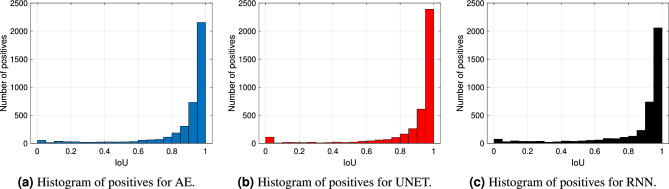
Histogram of detected USV calls (positives) with respect to the corresponding IoU value with post-processing.

Thus, Fig. 7a,b show the precision and the recall curves associated with the models at varying of the considered IoU threshold. As before, the solid lines correspond to the segmentation performance when the post-processing has not been applied. Those curves show high recall, but low precision, values. This indicates that some noisy spikes can be wrongly detected outside of a true USV call. As expected, such spikes increase the number of FP, affecting this way the precision of the models. On the contrary, the implementation of the post-processing algorithm allows to remove this type of wrong detection. In fact, both the recall and, especially, the precision curves associated with the post-processing algorithm (dashed lines) present a strong improvement. Fig. 8 also shows the histogram of the detected USVs (positives) with respect to the corresponding IoU value, which is significantly squeezed over IoU values greater than 0.8, thus proving the efficiency of the methods. Overall, the UNET architecture outperforms both the AE and the RNN models, with the latter returning the lowest performance even if characterized by the largest number of parameters.

**Figure 9:**
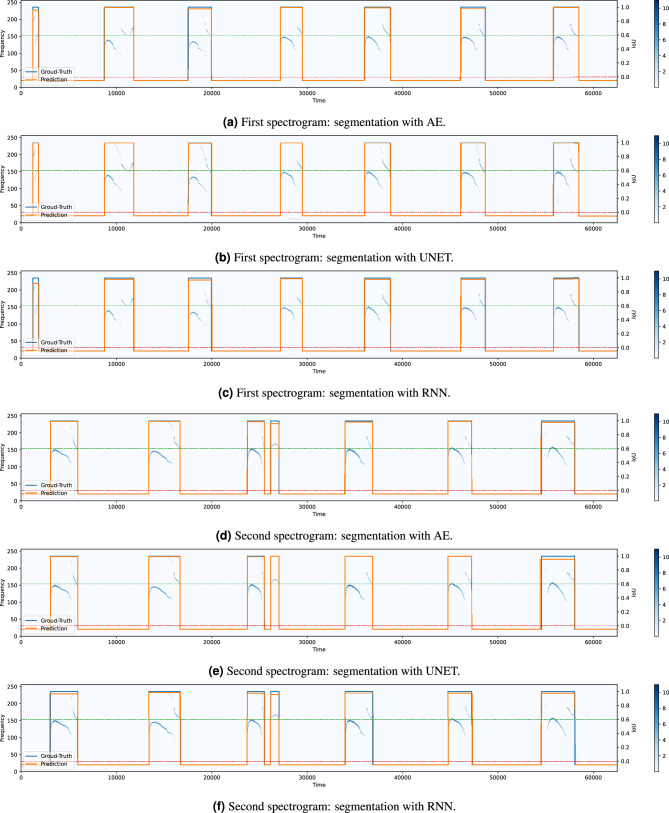
Visual results comparison between the proposed models with the post-processing algorithm. The pictures show the spectrograms of two audio segments (the first segment is depicted in (**a**– **c**) while the second one in (**d**–**f**) with the associated GT (blue signal) and prediction (orange signal) related to the three models.

To better appreciate the quality of the results, in Fig. 9 some visual results are illustrated. In particular, Fig. 9a–c and Fig. 9d–f show the segmentation resulting from the three proposed methods applied to two different spectrograms associated with two audio tracks. The blue and orange signals represent the GT and the prediction, respectively. The GT assumes just two values, 1 and 0, depending on the presence or not of a vocalization. To remark the quality of the detection, the amplitude of the prediction signal is set equal to either the corresponding IoU value (from 0 to 1) when a USV has been detected or $-0.5$ when, correctly, no USV has been found. To improve clarity, the IoU values 0 (related to zero intersection between GT and prediction) and 0.6 (threshold adopted in this work) are highlighted with a dashed line with color red and green, respectively.

### Extended comparison

In order to further evaluate the validity of the proposed architectures, their performance have been compared with the performance of 4 alternative methods: DeepSqueak^22^ (DS), HybridMouse^25^ (HM), A-MUD^18^ and USVSEG^19^. DS has been proposed as the first vocalization segmentation and classification tool based on an artificial neural network, and it represents one of the state-of-the-art methods in literature for USV segmentation. In our comparison, only the segmentation part of the software will be considered. Note that it also offers the possibility to the user to review each segmented output indicating if it is a real USV (true positive) or a wrong detection (false positive). This manual review can be used to train an ad-hoc de-noising network in order to refine the first segmentation. Alternately, HM is a more recent ultrasonic audio analysis tool which combines convolutional and recurrent neural networks for automatically identifying, labeling and extracting recorded USVs, and showed very high performance. In contrast, A-MUD and USVSEG rely on signal processing analysis, prioritizing low complexity even if parameter settings are necessary.

Table 1 presents the performance comparison between our proposed models with post-processing (when setting a threshold of $t_c=0.5$, see Fig.5b) and the competitive methods in correctly classifying individual time frames. The evaluation metrics include precision, recall, accuracy, and False Positive Rate (FPR), defined as $\text {FPR}=\frac{\text {FP}}{\text {FP}+\text {TN}}$. Also the evaluations of the competitive methods have been obtained by testing all audio tracks in our test set. As it was observed in the previous section, the three proposed models present very high performance, with the precision, recall and accuracy values greater than $90\%$ for all the three methods. Furthermore, FPR is consistently smaller than $0.8\%$. Overall, our models consistently outperform the competitive methods, with the exception of precision, where A-MUD achieves an accuracy of $98\%$.

Next, Table 2 reports the comparison regarding the USV segmentation performance in terms of precision and recall. Note that for DS, we have also reexamined its outputs for a subset of the dataset (one mouse per each cage) and used them for training the de-noising network. Then, the trained de-noising network has been applied on the DS outputs of the whole test set. As expected, the de-noising network has been able to discard some noisy segments, as proven by the increase of the precision, while the recall is unchanged since the network does not fix potential false negatives. Regarding the results presented in the table, they indicate that both AE and UNET achieve precision and recall rates exceeding $90\%$. These two models, along with RNN, outperform DS and HM, even when the denoising network is employed alongside the DS segmentator. This achievement is particularly notable given that UNET, which exhibits the best results, comprises only 187, 113 parameters-approximately a quarter of the parameters utilized in DS (693, 320) and two-thirds of the parameters in HM (283, 300). Additionally, both AE and UNET outperform A-MUD and USVSEG in terms of recall, while A-MUD achieves the same precision performance as AE, with UNET still demonstrating slightly superior performance .

**Table 1:** Precision, recall, accuracy and FPR values for time frame classification obtained on the test set of our dataset.

	AE	UNET	RNN	A-MUD	DS	USVSEG	HM
Precision	95.62%	95.38%	**95.97%**	*98.13%*	95.79%	95.47%	76.85
Recall	**95.46%**	*97.14%*	92.10%	84.59%	81.92%	94.41%	94.95%
Accuracy	**99.26%**	*99.35%*	99.04%	98.39%	98.02%	99.05%	98.39%
FPR	**0.35%**	*0.28%*	0.78%	0.37%	0.36%	0.46%	2.98%

Comparison between AE, UNET, RNN, A-MUD, DS, USVSEG and HM. $t_c = 0.5$ for our methods. The best performer is marked in italic, while the second best performer is highlighted in bold for the two metrics.

**Table 2:** Precision and recall values for USV segmentation obtained on the test set of our dataset, considering $t_{\text {IoU}} = 0.6$. Comparison between AE, UNET, RNN, A-MUD, DS, DS with de-noiser, USVSEG and HM. $t_c = 0.5$ for our methods.

	AE	UNET	RNN	A-MUD	DS	DS (w/ de-noiser)	USVSEG	HM
Precision	90.14%	*91.11%*	86.18%	**90.62%**	66.37%	75.75%	85.72%	82.03%
Recall	**90.80%**	*92.08%*	84.15%	80.03%	63.72%	63.66%	87.99 %	71.18%

The best performer is marked in italic, while the second best performer is highlighted in bold for the two metrics.

**Table 3:** Precision and recall values for USV segmentation obtained on the external dataset, considering $t_{\text {IoU}} = 0.6$. Comparison between AE, UNET, RNN, A-MUD, DS, DS with de-noiser, USVSEG and HM. $t_c = 0.5$ for our methods.

	AE	UNET	RNN	A-MUD	DS	DS (w/ de-noiser)	USVSEG	HM	
Precision	*66.33%*	**65.03%**%	57.95%	49.21%	60.91%	62.00%	51.60%	57.86%	B6pup
Recall	65.81%	66.81%	55.23%	47.41%	68.71%	**68.75%**	*69.64%*	58.46%	
Precision	84.14%	*90.52%*	**85.58%**	77.52%	61.40%	62.76%	69.40%	70.89%	BALB/c
Recall	**77.12%**	*80.45%*	73.38%	48.25%	54.05%	53.84%	76.64%	56.40%	
Precision	70.65%	77.71%	76.87%	77.41%	**83.15%**	*84.70%*	66.56%	56.94%	C57BL/6J
Recall	64.91%	**71.77%**	66.60%	62.40%	61.18%	61.18%	*78.12%*	46.98%	
Precision	69.20%	**70.02%**	40.85%	23.48%	53.61%	53.61%	44.78%	*75.85%*	Shank2-
Recall	*78.13%*	**73.33%**	39.69%	16.73%	57.94%	57.05%	65.02%	71.53%	
Precision	**70.70%**	*74.25%*	68.03%	64.50%	61.19%	62.25%	60.68%	60.18%	Overall
Recall	68.34%	**69.05%**	62.99%	50.77%	56.73%	56.43%	*74.87%*	53.50%	

The best performer is marked in italic, while the second best performer is highlighted in bold for the two metrics.

Finally, to assess the applicability of our procedure for a wider range of strains and situations, we have also validated our algorithms on a different variegate dataset, which was partially collected in^19^. The audio files with the related labels have been downloaded from http://doi.org/10.5281/zenodo.3428024: in total, 5, 2, 10 and 3 audio tracks recorded during C57BL/6J pups maternal isolation (B6pup), BALB/c male courtship, C57BL/6J male courtship and Shank2- male courtship, respectively, have been analyzed. For details about Shank2- mice, that is a model of autism spectrum disorders, see^30^. The audio tracks vary in duration, ranging from 53 seconds to 206 seconds. The time samples within the tracks are distributed between USV and noise, with a ratio of approximately 1 to 10. Specifically, there are 751, 733, 518 noise samples and 85, 570, 802 USV samples. Within the entire dataset, there are a total of 4, 048 USVs, which serve as the ground-truth.

Thus, Table 3 reports the USV segmentation performance associated with our models (without re-training), as well as the ones associated with the competitive methods, for this different dataset. Table S1 in the supplementary material also provides the results regarding the time frame classification. To give a comprehensive analysis, we present both the overall performance and the performance associated with each specific strain. The precision and recall values of our proposed methods are slightly decreased due to the diversity of the data compared to the data used for training our models. However, both AE and UNET consistently maintain precision and recall rates above $65\%$ for every strain, unlike the other methods where the performance notably declines in at least one strain. This serves as evidence of the robustness of AE and UNET. In terms of precision, AE and UNET achieve the top two values, with UNET surpassing $74\%$. Regarding recall, USVSEG achieves the highest result at approximately $74\%$, while UNET follows closely with around $69\%$. It is important to acknowledge that there might be a potential bias in favor of USVSEG for this dataset. Indeed, USVSEG requires the configuration of certain parameters, and in our experiments, we employed the optimal parameters recommended in the original paper^19^. It is worth noting that USVSEG was specifically trained on tracks of C57BL/6J mice, which is, in fact, the only strain where USVSEG demonstrates a substantial performance advantage over UNET, as depicted in Table 3.

It should be noted that the spectrograms of the audio data in this dataset have been generated using the same configuration settings for obtaining the spectrograms of the audio tracks of our dataset. However, this has resulted in spectrograms with a generally lower signal-to-noise ratio, possibly due to variations in the recording procedures. This discrepancy may be one of the reasons for the overall lower performance observed. Nevertheless, this aspect allows us to appreciate the behavior of our models and the competitive ones, even when dealing with spectrograms that exhibit variations in the intensity of the USV calls.

### Analysis on detected USV lengths

**Figure 10:**
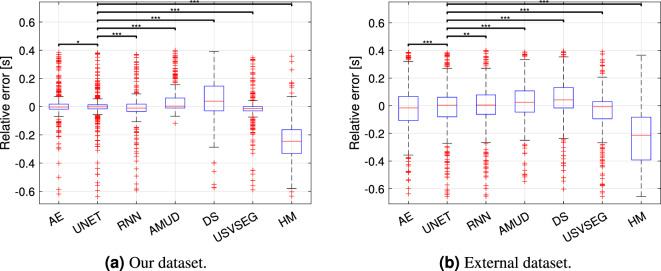
Boxplots related to the length relative errors distributions.

In this Section, we aim to investigate potential differences in the duration of the USVs detected by our proposed models and the previously compared competitive models. To accomplish this, we have computed the relative error $$(l_\text {GT} - l_\text {pr})/l_\text {GT}$$ for each correctly segmented USV across all methods, in both datasets. Thus, $$l_\text {pr}$$ is a vector in which each element represents the length of a TP USV, while $$l_\text {GT}$$ is a vector in which each element represents the corresponding true length. Fig. 10a,b illustrate the boxplots depicting the distribution of the relative errors of the models for our dataset and the external dataset, respectively. For our dataset, AE and UNET exhibit a median relative error close to zero, indicating a high level of accuracy with a minimal spread. Conversely, for the external dataset, UNET, RNN, and USVSEG appear to be the most accurate methods, although with a wider spread compared to our dataset. In order to determine whether there are significant differences in the estimation of syllable durations among the methods, we have conducted the Wilcoxon rank sum test ($$*p<0.05$$, $$**p<0.01$$, $$***p<0.001$$), specifically comparing the distributions of relative errors between UNET and the other methods (see Fig. 10). For the sake of completeness, in Fig. S1 in the supplementary material, we also show the one-to-one comparison between all the lengths of the predicted USVs and the true lengths for each model and dataset.

## Conclusions

In this paper, the mice ultrasonic vocalization segmentation task has been faced comparing an Auto-Encoder Neural Network (AE), a U-NET Neural Network (UNET) and a Recurrent Neural Network (RNN). To assess the performance of the models, we have created a dataset by recording several audio tracks and manually segmenting the corresponding USV spectrograms. As a preliminary analysis, we focused on the ability to detect the presence of a USV for each single time frame, without considering the final identification of a vocalization. In this case, all the three proposed architectures returned precision and recall scores greater than $90\%$, with the UNET and the AE which reporting values superior than $95\%$. When studying the performance related to the USV segmentation, the results remain significantly high. In fact, the precision and recall scores for the UNET and the AE are larger than $90\%$, while the values for the RNN are around $85\%$.

Such performance also overcome the one associated with popular software tools for USV segmentation, such as A-MUD, DeepSqueak (DS), USVSEG, and HybridMouse (HM). Furthermore, the validation has been expanded also to an external dataset. Overall, the UNET is the model reporting the highest performance, thus outperforming both the AE and the RNN. Noticeably, the 3 proposed models are characterized by a quite small number of parameters (especially the AE and the UNET), thus their use is also particularly efficient from a computational point of view.

In future endeavors, we plan to integrate our proposed models into a USV segmentation-classification system. This integration will provide an opportunity to explore the correlation between enhanced segmentation accuracy and its subsequent influence on achieving improved classification outcomes.

## Supplementary Information

Supplementary Information.

## Data Availability

The data and the software that support the findings of this study are publicly available at https://osf.io/8sgkh/?view_only=4cb73a92e666485382696e410abd75a2.
