# Supplementary material for: Extended performance analysis of deep-learning algorithms for mice vocalization segmentation
Source: Sci Rep. 2023 Jul 11;13:11238. doi: 10.1038/s41598-023-38186-7 (PMC10336146; doi:10.1038/s41598-023-38186-7)
Supplement: Supplementary file 1 — Supplementary Information. [file 41598_2023_38186_MOESM1_ESM.pdf]

Extended performance analysis of deep-learning algorithms for mice vocalization segmentation - Supplementary material

Daniele Baggi[1], Marika Premoli[2], Alessandro Gnutti[1,*], Sara Anna Bonini[2], Riccardo Leonardi[1], Maurizio Memo[2], and Pierangelo Migliorati[1]

[1]Department of Information Engineering, University of Brescia, Brescia, Italy
[2]Department of Molecular and Translational Medicine, University of Brescia, Brescia, Italy
[*]alessandro.gnutti@unibs.it

	AE	UNET	RNN	A-MUD	DS	DS (w/ de-noiser)	USVSEG	HM	
Precision	73.93%	75.90%	63.37%	96.55%	92.45%	92.54%	91.97%	75.32%	B6pup
Recall	94.03%	92.18%	93.83%	52.10%	69.75%	69.64%	77.15%	87.03%	
F1	82.78%	83.25%	75.65%	67.68%	79.51%	79.47%	83.91%	80.75%	
FPR	0.40%	0.37%	0.57%	0.03%	0.09%	0.08%	0.11%	0.46%	
Precision	93.49%	92.01%	82.67%	96.44%	93.44%	93.44%	92.09%	75.75%	BALB/c
Recall	85.58%	90.20%	92.33%	50.57%	72.24%	72.24%	86.30%	90.52%	
F1	89.36%	91.10%	87.24%	66.35%	81.48%	81.48%	89.10%	82.48%	
FPR	0.71%	0.86%	1.84%	0.21%	0.54%	0.54%	0.83%	3.26%	
Precision	97.93%	97.45%	95.38%	90.29%	90.18%	90.23%	87.29%	73.48%	C57BL/6J
Recall	83.64%	86.89%	88.67%	50.03%	70.69%	70.62%	97.50%	97.64%	
F1	90.23%	91.87%	91.90%	64.38%	79.25%	79.23%	92.11%	83.85%	
FPR	0.20%	0.25%	0.45%	0.56%	0.76%	0.75%	1.42%	3.68%	
Precision	87.26%	83.99%	59.04%	99.99%	98.21%	98.27%	98.45%	89.28%	Shank2-
Recall	94.81%	97.40%	99.62%	31.30%	56.75%	56.38%	77.12%	87.63%	
F1	90.88%	90.20%	74.14%	47.67%	71.93%	71.65%	86.49%	88.45%	
FPR	4.23%	0.52%	12.22%	0.01%	0.35%	0.33%	0.41%	3.61%	
Precision	90.21%	90.84%	83.18%	92.92%	92.36%	92.31%	90.26%	77.24%	Overall
Recall	87.07%	90.14%	90.34%	45.09%	66.90%	66.75%	89.73%	93.68%	
F1	88.61%	90.49%	86.61%	60.72%	77.59%	77.48%	90.00%	84.67%	
FPR	0.58%	0.67%	1.45%	0.29%	0.46%	0.45%	0.82%	2.38%	

Table S1. Precision, recall, F1 and FPR scores for time frame classification obtained on the external dataset. Comparison between AE, UNET, RNN, A-MUD, DS, DS with de-noiser, USVSEG and HM. $t_c = 0.5$ for our methods. The best performer is marked in red, while the second best performer is highlighted in blue for the two metrics.

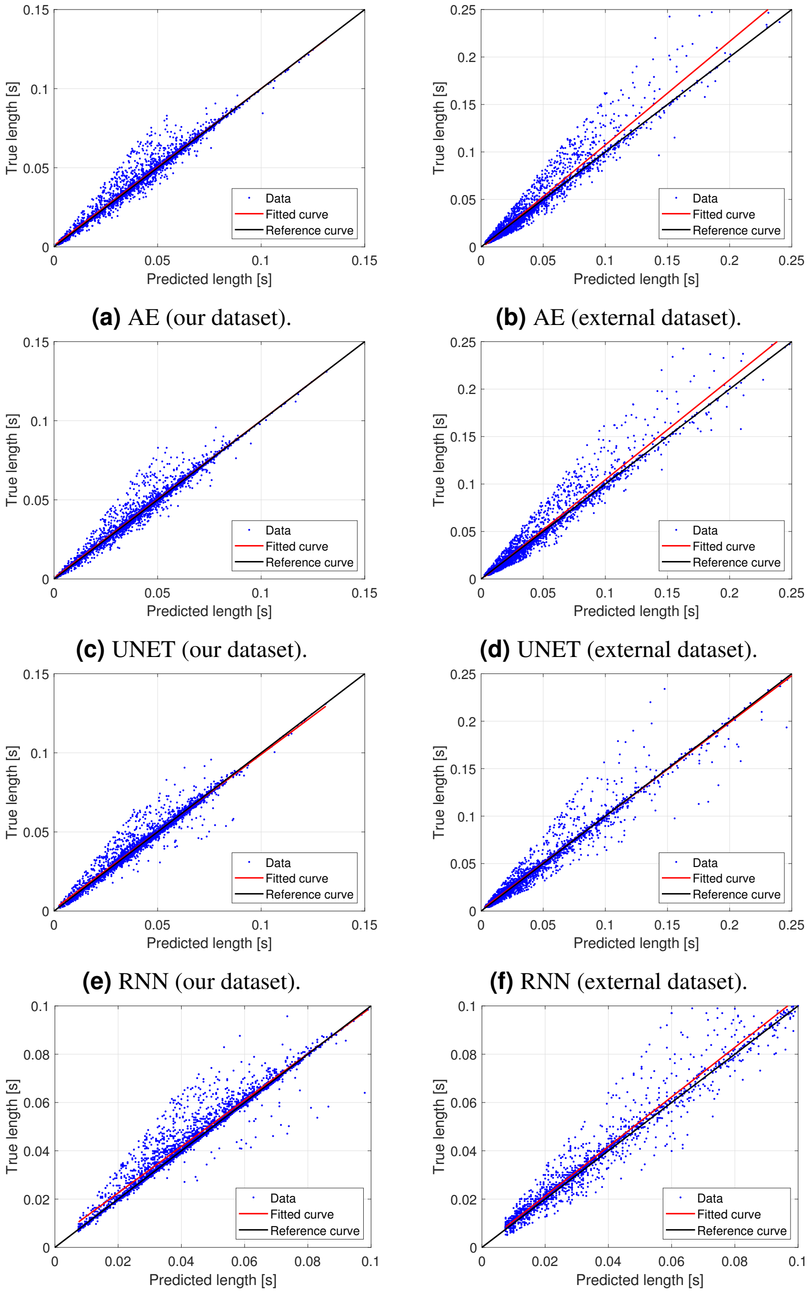

(a) AE (our dataset).

(b) AE (external dataset).

(c) UNET (our dataset).

(d) UNET (external dataset).

(e) RNN (our dataset).

(f) RNN (external dataset).

(g) A-MUD (our dataset).

(h) A-MUD (external dataset).

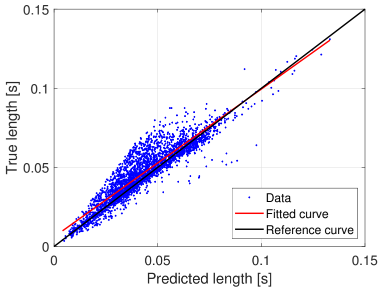

(i) DS (our dataset).

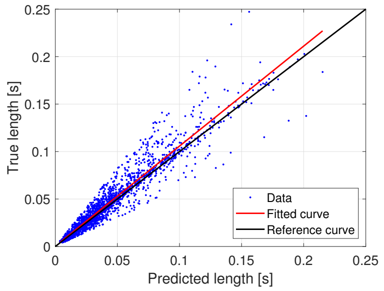

(j) DS (external dataset).

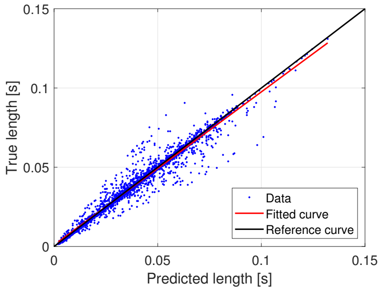

(k) USVSEG (our dataset).

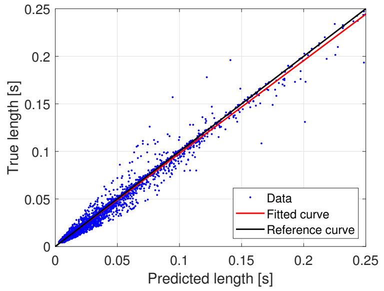

(l) USVSEG (external dataset).

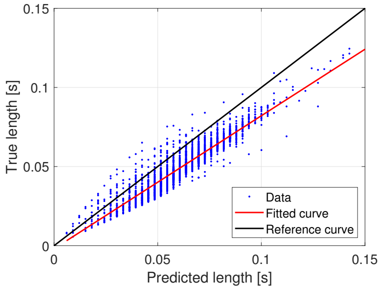

(m) HM (our dataset).

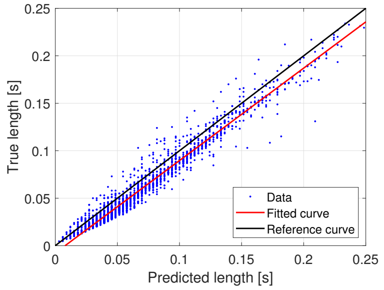

(n) HM (external dataset).

Figure S1. The blue points represent the data points, while the red line represents the curve that best fits the point cloud. Ideally, this curve would align with the bisector of the plane, indicated as the reference line in black. The regression lines corresponding to our dataset for AE, UNET, and RNN align almost perfectly with the reference lines. Although AE and UNET exhibit a slight decline in performance on the external dataset, the regression line of RNN closely follows the reference line even on the external dataset. USVSEG demonstrates good accuracy on the external dataset. In contrast, HM consistently tends to estimate a longer duration for the USVs compared to their true extent.
